# Can Routine Patients Be Safely Discharged After Neodymium-Doped Yttrium Aluminium Garnet Laser Posterior Capsulotomy?

**DOI:** 10.7759/cureus.21803

**Published:** 2022-02-01

**Authors:** James G Richardson-May, Chuiki J La, Madalina A Chihaia, Holly Clarke, Michelle Light, Mohammed Rashid

**Affiliations:** 1 Ophthalmology, University Hospital Southampton NHS Foundation Trust, Southampton, GBR; 2 Ophthalmology, University Hospitals Dorset NHS Foundation Trust, Bournemouth, GBR; 3 Ophthalmology, Queen Alexandra Hospital, Portsmouth, GBR; 4 Medical Statistics, University Hospital Southampton NHS Foundation Trust, Southampton, GBR

**Keywords:** cataract patients, general ophthalmology, laser treatment, fibrous capsule, yag laser capsulotomy

## Abstract

Background

Neodymium-doped yttrium aluminium garnet (Nd:YAG) posterior capsulotomy is a common treatment for posterior capsular opacification. Practice varies regarding routine follow-up. In this study, we reviewed follow-up rates and treatment-related complications from a district general hospital’s ophthalmology unit to assess areas for improvement and cost-effectiveness.

Methodology

We conducted a retrospective review of electronic patient records for all patients treated with Nd:YAG capsulotomy in 2019 at our hospital. Primary outcomes included visual acuity, complications, and follow-up data. Secondary outcomes included medication prescribing and the grade of surgeon.

Results

In total, 912 eyes of 744 patients were included. Overall, 536 (58.8%) eyes were discharged immediately following their laser. Complication rate was 4.3% (39 eyes). Junior training grades had a higher rate of medication prescribing (40/46 eyes; 87.0%) and follow-up (36/40 eyes; 78.3%).

Conclusions

Certain selected patients may be safely discharged following capsulotomy with safety-netting advice. This strategy increases the capacity to follow-up patients at higher risk of complications. Higher rates of follow-up among junior ophthalmologists offers potential for training.

## Introduction

Neodymium-doped yttrium aluminium garnet (Nd:YAG) posterior capsulotomy (YAG-PC) is a common elective outpatient laser procedure for the treatment of posterior capsular opacification (PCO). PCO is one of the most common complications of cataract surgery and represents a significant proportion of referrals to secondary ophthalmology services. A retrospective analysis published in *Eye* reported that 5.8-19.3% of pseudophakic patients underwent YAG-PC treatment across seven different eye units in the United Kingdom within five years of their cataract surgery [1].

We anecdotally noted a large number of patients being reviewed routinely in the outpatient department following their laser procedure, despite no other ocular co-morbidities and an uncomplicated procedure. Currently, there is a lack of consensus regarding routine follow-up practice after YAG-PC, with very limited published data. A survey of 132 UK-based National Health Service (NHS) consultant ophthalmologists in 2011 reported that 40% of respondents routinely follow-up their patients after YAG-PC [2]. A recent survey in EyeNews reported that 22.1% of respondents routinely followed up their patients after YAG-PC [3]. In a climate where backlogs for ophthalmology outpatient appointments are ever-expanding, we aimed to review follow-up and complication rates following YAG-PC treatment in our unit to reveal any potential scope for direct discharge following treatment, thereby increasing capacity for outpatient appointments.

## Materials and methods

Electronic patient records were retrospectively reviewed for all patients who underwent YAG-PC between 1st January 2019 and 31st December 2019 at the Royal Bournemouth Hospital in the United Kingdom. Follow-up data were collected from 1st January 2019 up to 30th April 2020, when note review began. Primary outcomes included best-corrected visual acuity (BCVA) before and after laser and intra-operative and post-operative complications. Complications were defined as unexpected ocular findings that could be attributed to the laser procedure. Secondary outcomes included the grade of surgeons, ocular co-morbidities (concurrent ocular pathology with a significant impact on visual function), and post-procedure medication prescribing. Records for planned and unplanned follow-up appointments in both the outpatient department and eye emergency clinic were reviewed. Because individual eyes for patients are often treated on separate occasions, we will refer to these instances as “eyes” as opposed to “patients” because different eyes may have different outcomes.

## Results

Follow-up

In total, 912 eyes of 744 patients were identified who underwent the laser procedure in 2019. Overall, 536 of 912 (58.8%) eyes were discharged immediately following their laser, while 339 (37.2%) had arranged follow-up, and 20 (2.19%) attended for unplanned review at the eye emergency department. In total, 35 (3.84%) eyes were lost to follow-up, and two (0.22%) did not attend their planned follow-up. Six (0.66%) eyes had follow-up planned in the future, which had been delayed due to the coronavirus pandemic.

Complications

The overall post-procedure complication rate was 4.3% (n = 39). Of the 339 eyes with planned follow-up, 23 had post-procedure complications (6.8%; see Table 1); of these, one was identified following attendance to the eye casualty. Three patients with post-operative complications were subsequently discharged at their follow-up visit. Overall, 262 (69.7%) eyes in this group had other ocular co-morbidities. In total, 152 (44.84%) eyes from this group were discharged at their follow-up appointment.

**Table 1:** Post-operative complications in eyes with planned follow-up deemed related to posterior capsulotomy. May have multiple findings in individual eyes. AMD: age-related macular degeneration

Complication	Number	Identified at planned follow-up
Wet AMD reactivation	2	2
Anterior uveitis	3	3
Cystoid macular oedema	8	7
Diabetic macular oedema	1	1
Corneal oedema	1	1
Floaters	4	2
Narrow capsulotomy	3	3
Recurrence of herpes simplex keratitis	1	1
Total	23	

Of the 536 eyes that were discharged directly following their laser, 54 (10.07%) had other ocular co-morbidities (Table 2). Moreover, 16 (3.0%) eyes had complications post-procedure (Table 3), which were identified following the patients’ self-referral to eye casualty. Of these, two needed further follow-up (one had rhegmatogenous retinal detachment, while another had posterior vitreous detachment which was subsequently referred for contralateral YAG-PC). The rate of more minor, non-sight-threatening problems was higher in this group; eight complained of floaters, two had subconjunctival haemorrhage, one had dry eye, and one had issues relating to their drops. In total, 49 eyes had an intra-procedure complication recorded, including one subconjunctival haemorrhage and 48 with pitting of the lens.

**Table 2:** Co-morbidities in patients with planned follow-up and those directly discharged after their laser procedure. Individual eyes may have more than one co-morbidity.

Co-morbidity	Number (planned follow-up group)	Number (discharged from laser group)
Retinal		
Dry age-related macular degeneration	53	14
Wet age-related macular degeneration	24	1
Retinal vein occlusion – branch	4	
Retinal vein occlusion – central	7	
Retinal artery occlusion – branch	1	
Retinal artery occlusion – cilioretinal	1	
Cystoid macular oedema (non-diabetic)	11	
Diabetic retinopathy – non-proliferative	3	4
Diabetic retinopathy – proliferative	6	1
Diabetic macular oedema	6	
Vitreous haemorrhage		1
Toxoplasmosis chorioretinitis	2	
Previous endophthalmitis	1	
Retinitis pigmentosa	1	
Choroidal naevus	1	
High myopia	6	4
Myopic choroidal neovascularisation	1	
Previous retinal detachment (including surgery)	17	6
Macular/lamellar hole (including surgery)	9	4
Epiretinal membrane (including surgery)	31	6
Proliferative vitreoretinopathy (including surgery)	1	
Retinal dialysis	1	
Retinoschisis		1
Retinal tear		1
Glaucoma		
Type not specified	15	
Ocular hypertension	9	1
Glaucoma suspect	7	
Primary open-angle glaucoma	39	3
Normal-tension glaucoma	7	1
Acute angle-closure glaucoma	2	
Rubeotic glaucoma	1	
Filtration surgery	10	
Pigment dispersion syndrome	1	
Anterior segment + cornea		
Dry eyes, Meibomian gland dysfunction, blepharitis	6	1
Corneal graft	3	
Complicated cataract surgery	1	
Fuchs’ endothelial dystrophy	7	1
Herpes simplex keratitis	3	
Conjunctival lesion excision	1	
Corneal dystrophy	1	
Corneal scar	2	
Acanthamoeba	1	
Herpes zoster ophthalmicus	1	
Pterygium		1
Band keratopathy		1
Anterior uveitis	6	
Neurological		
Amblyopia	6	1
Anterior ischaemic optic neuropathy	2	
Optic atrophy	2	
Visual field defect (cause not specified)	2	
Other		
Ectropion	2	
Nasolacrimal duct obstruction	1	
Squint	1	
Total	325	54

**Table 3:** Post-operative complications in eyes discharged following laser deemed related to posterior capsulotomy. May have multiple findings in individual eyes. All were identified at unplanned visits to the eye casualty department. PVD: posterior vitreous detachment; IOP: intraocular pressure

Complication	Number
Floaters	8
PVD	2
Rhegmatogenous retinal detachment	1
Subconjunctival haemorrhage	2
Drop-related problem	1
Dry eye	1
IOP spike	1
Total	16

Post-operative medication

In total, 568 (62.28%) patients had no topical medications following their laser; of these, 15 (2.64%) had complications relating to their procedure. Overall, 337 (36.95%) patients received topical medications post laser, of whom 24 (7.12%) had complications. Moreover, 123 (36.5%) of these patients had topical steroids, 20 (5.9%) a non-steroidal anti-inflammatory drug (NSAID), and 194 (57.6%) had both steroid and NSAID. Post-operative complications were observed in seven (5.7%), one (5.0%) and 16 (8.2%) of steroid, NSAID, or steroid and NSAID users, respectively. The odds ratio for those who had complications in the group without medication versus those who did was 0.37 (95% confidence interval = 0.19-0.72).

Best-corrected visual acuity

Table 4 demonstrates visual acuity before and after the procedure. Post-procedure BCVA was available for 347 eyes of those who returned to the eye unit. In total, 206 (59.37%) improved following their laser, 95 (27.38%) had the same BCVA, and 46 (13.26%) were worse. Of these, 29 lost one line on Snellen acuity, 15 lost two lines, and one each lost three or four lines. In most cases, the final BCVA was improved including those who suffered complications following their YAG-PC. In total, 22 of 39 (56.41%) eyes showed improvement in BCVA, three (7.69%) had no change, and 10 (25.64%) had worsening of their BCVA. Of these 10 eyes, six lost one line of Snellen acuity, three lost two lines, and one lost four lines of acuity. The remaining four patients had no acuity recorded to compare.

**Table 4:** Visual acuity before laser and at follow-up (if recorded). CF: count fingers; HM: hand movements; PL: perception of light

Visual acuity	Pre-procedure	Post-procedure
Not recorded	12 (1.31%)	4 (1.15%)
6/4 to 6/7.5	186 (20.39%)	137 (39.48%)
6/9 to 6/12	346 (37.94%)	123 (35.45%)
6/15 to 6/38	274 (30.04%)	60 (17.29%)
6/48 to 6/60	31 (3.40%)	1 (0.29%)
CF to HM	59 (6.47%)	20 (5.76%)
PL	4 (0.44%)	2 (0.58%)

Grade of performing ophthalmologist

Procedures were undertaken by a range of grades of ophthalmologists (Table 5), with most being performed by non-training staff grades (367, 40.24%). More junior grades (ST1-3) had a higher rate of medication prescribing (40/46, 86.96%) and follow-up (36/46, 78.26%) than their senior counterparts. Consultants, associate specialists, non-training specialist registrars, and specialty training registrars (ST) year four to seven had a lower follow-up rate (54/145, 51/109, 12/89, 35/156; 37.24%, 46.79%, 13.48%, 22.44%, respectively) and medication prescribing rate (26/145, 30/109, 3/89, 10/156; 17.93%, 27.52%, 3.37%, 0.06%, respectively). Complications rates were similar between grades: 2/46 (4.35%) ST1-3; 4/156 (2.56%) ST4-7; 2/89 (2.25%) non-training registrar grade; 20/367 (4.45%) staff grade; 4/109 (3.67%) associate specialist, and 7/145 (4.83%) consultant.

**Table 5:** Data grouped by the grade of surgeons.

Grade	Number of procedures	Medication prescription	Eyes for routine follow-up	Eyes discharged after laser	Lost to follow-up	Complications
Associate specialist	109 (11.95%)	30 (27.52%)	51 (46.79%)	56 (51.38%)	2 (1.83%)	4 (3.67%)
Consultant	145 (15.90%)	26 (17.93%)	54 (37.24%)	84 (57.93%)	8 (5.52%)	7 (4.83%)
Staff grade	367 (40.24%)	228 (62.13%)	156 (42.51%)	193 (52.59%)	17 (4.63%)	20 (4.45%)
SpR (non-training)	89 (9.76%)	3 (3.37%)	12 (13.48%)	77 (86.52%)	0	2 (2.25%)
ST1-3	46 (5.04%)	40 (86.96%)	36 (78.26%)	8 (17.39%)	2 (4.35%)	2 (4.35%)
ST4-7	156 (17.11%)	10 (0.06%)	35 (22.44%)	114 (73.08%)	7 (4.49%)	4 (2.56%)
Total	912	337	344	532	36	39

## Discussion

YAG-PC is considered to be a safe and effective procedure for the management of PCO. Despite this, we have a relatively high rate of patients being followed up routinely after their procedure with inconsistent post-operative care among our staff cohort. Of the 339 eyes with planned follow-up after their laser, 152 (44.84%) were discharged at their follow-up due to an uneventful post-treatment course. We suggest that these patients could have been safely discharged at their laser clinic visit, reducing the burden on outpatient appointments as well as reducing hospital attendances for the patient; this is particularly poignant in the current climate of coronavirus disease 2019 (COVID-19). Of note, routine follow-up is acknowledged but not required in the previous guidelines from the Royal College of Ophthalmologists. These guidelines recommend an advice sheet should the patient not be followed-up [4,5].

A survey in EyeNews in 2020 found that 22.1% of respondents routinely followed up their patients after YAG-PC. This is lower than our rate of 37.17%. Moreover, 41% routinely gave drops after laser capsulotomy (higher than our rate of 36.95%), of whom 74.6% gave steroids and 20.5% gave NSAIDs [3]. Another survey published in the *European Journal of Ophthalmology* in 2011 reviewed the practice of 132 UK-based NHS consultant ophthalmologists. The study highlighted that 60% of those surveyed do not follow up their patients routinely. Moreover, 42.4% routinely gave post-operative steroid drops [2] compared to 34.8% in our hospital.

Complication rates in our patients were 3.0% in the eyes discharged directly from their laser appointment and 6.8% in those who had planned follow-up. The odds of complications was significantly reduced in those discharged directly from the laser (odds ratio = 0.44; 95% confidence interval = 0.23 to 0.84). Of those discharged directly, all patients suffering complications self-presented to the eye emergency department, as suggested, for diagnosis and management of their complications. Many complications were relatively minor, with little or no long-term visual significance; these complications included dry eye, subconjunctival haemorrhage, and an increase in vitreous floaters with no evidence of retinal injury. We did, however, identify a small number of patients with visually significant complications, including cystoid macular oedema and retinal detachment.

The rate of ocular co-morbidity was higher in patients brought back for follow-up. Overall, 69.67% of those with arranged follow-up had other ocular diagnoses versus 10.07% of those who were discharged. The group of patients who underwent planned follow-up had a higher rate of complications, such as cystoid macular oedema, recurrence of herpes simplex keratitis, reactivation of wet age-related macular degeneration, and anterior uveitis. This would be expected given the patient population and represents appropriate follow-up for this cohort of patients. In those who had complications, the rate of co-morbidity was lower in the group discharged after laser treatment (4 of 16 eyes, 25.0%) than those who had planned follow-up (22 of 26, 84.6%).

Reassuringly, BCVA improved or remained stable in the majority of patients (59.37% improved, 27.38% stable), including those with subsequent complications (56.41% improved, 7.69% stable). BCVA was unavailable for many patients, the majority of whom were discharged from their laser appointment and thus did not reattend the eye unit.

The grade of clinicians performing YAG-PC influenced follow-up rates as well as medication prescribing rates. Junior training grades (ST1-3) had a higher follow-up rate (78.26%) and medication prescribing rate (86.96%) compared to their senior counterparts (consultants, associate specialists, non-training specialist registrars, and ST4-7 trainees). The vast majority of our YAG-PCs were undertaken by staff grade clinicians, with a follow-up rate of 42.51% and prescribing rate of 62.13%. The complication rate varied slightly between the grade of clinicians; 4.35% for ST1-3, 2.56% for ST4-7, 2.25% for non-training registrars, 4.45% for staff grade, 3.67% for associate specialists, and 4.83% for consultants. The complication rate was slightly higher in the group who received post-procedure medication, though this may reflect patients with a more pro-inflammatory capsulotomy.

It is not surprising that more junior staff are more likely to bring patients back for follow-up. This may reflect a lack of confidence in discharging patients. Patients referred by their optometrists are often booked directly into the laser clinic, and therefore, have not seen another clinician prior to their treatment. It may also reflect a “trickle-down” effect from their trainer; with a lack of formal guidance, if the clinician who taught the junior laser followed up their patients, the junior is more likely to do so too. It is also unsurprising that they were more likely to prescribe medication, being unaware that many YAG-PCs raise very mild inflammatory responses. Persistent iritis rates are estimated at 0.4-1.4% [6,7].

We would suggest that discharging select patients directly following their laser procedure, with advice to self-present to the eye emergency department, would be a suitable approach that does not appear to increase the rate or severity of complications. Complications rates were slightly higher in patients who received post-operative drops (7.1% vs. 2.6%). Of those who had visually significant complications such as macular oedema, all had potential inciting causes, such as previous retinal vein occlusion, diabetic macular oedema, or wet macular degeneration. In most cases, it is likely to be safe to avoid routine prescription of anti-inflammatory medications. However, in patients with previous ocular issues (such as uveitis or diabetic eye disease) or those with a dense, “milky” posterior capsule, a short prescription of topical steroids is sensible. Of note, we have not included the rate of apraclonidine 1% drop use immediately pre and post-laser procedure as documentation of this varied widely. This may help to reduce the risk of intraocular pressure (IOP) spike after the procedure, which often occurs within the first hours. Hence, it seems pertinent to check IOP before discharging patients home, and we would suggest continuing the use of anti-hypertensives [4,8-10]. Unfortunately, IOP checks and ocular anti-hypertensive use was poorly documented for many of our patients, and therefore, we have been unable to formally assess their use. Anecdotally, the use of apraclonidine 1% immediately following the procedure is common in our institution.

There are several limitations to our work. This was a retrospective review of electronic records; a prospective study would allow standardisation of patients and more robust analysis. Documentation varied widely, and indeed made the assessment of IOP measurements and anti-hypertensive use difficult; this would be a useful topic to assess in the future. We have assumed that most patients would attend the local eye casualty department for any complications, but some may attend other hospitals, or not at all; these would not have been included in our analysis. Again, a prospective study would help address this shortfall.

Many complications occur either after the first few months following laser (such as retinal detachment and cystoid macular oedema) [4,11,12] or can be recognised at the time of the procedure or soon after (IOP spike, lens subluxation, hyphaema, pupil block, etc.) [4,8-10]. The former group may not have developed their complication by the time of their outpatient appointment, while the latter would occur prior to it and necessitate attendance at an eye emergency department regardless.

In our trust, consultant-led follow-up appointments attract a tariff of £58-£73. Using this as a guide, the follow-up visits at which 152 patients underwent uneventful discharge are likely to have accrued a cost of £8,816-£11,096. Furthermore, in the current era of COVID-19, reducing unnecessary face-to-face contact, as well as the burden on already stretched outpatient services, is a significant priority for secondary ophthalmic care.

## Conclusions

We do not dispute the need for patients undergoing YAG-PC with ocular co-morbidities to continue to undergo follow-up and drop therapy as the treating clinician deems appropriate. However, we suggest that uncomplicated patients can be safely discharged from their laser appointment with advice regarding when to present to eye emergency services in the event of a treatment-related complication. This advice should include warning signs for posterior vitreous or retinal detachment, as well as symptoms of acute IOP rise or inflammation such as pain or significant red eye. Further, adequate counselling before the procedure regarding symptoms to expect afterwards, such as some mild dry eye symptoms or a few floaters which should settle after a few days, will help to reduce unnecessary attendance and reduce patient anxiety. Details of whom to contact and where to attend both in- and out-of-hours should be included. Those with pre-existing ocular conditions, such as uveitis, herpetic disease, or macular oedema, are likely to benefit from a planned follow-up to monitor and treat for any worsening or reactivation.

We hope to add to a limited evidence base informing the current practice surrounding the routine follow-up of patients undergoing YAG-PC. Our suggested strategy offers the potential to alleviate a proportion of the current backlog crisis being experienced by eye units nationwide, without compromising patient safety. In the future, prospective studies may help bolster this currently limited area of evidence to further guide standard practice in a stretched health service.
